# Association between *PTEN* Gene IVS4 Polymorphism and Risk of Cancer: A Meta-Analysis

**DOI:** 10.1371/journal.pone.0098851

**Published:** 2014-06-05

**Authors:** Liping Sun, Jingwei Liu, Quan Yuan, Chengzhong Xing, Yuan Yuan

**Affiliations:** Tumor Etiology and Screening Department of Cancer Institute and General Surgery, the First Affiliated Hospital of China Medical University, and Key Laboratory of Cancer Etiology and Prevention (China Medical University), Liaoning Provincial Education Department, Shenyang, China; Wayne State University School of Medicine, United States of America

## Abstract

**Background:**

Phosphatase and tensin homolog (*PTEN*) is a well established tumor suppressor gene. Recently, increasing studies investigated the association between *PTEN* IVS4 polymorphism (rs3830675) and risk of various types of cancer. However, the results from the individual studies were controversial. The aim of this meta-analysis was to elucidate whether *PTEN* IVS4 polymorphism was associated with cancer risk.

**Methods:**

Databases including PubMed, Web of knowledge and Chinese National Knowledge Infrastructure (CNKI) were systematically searched to identify potentially eligible literatures. Odds ratios (OR) and their 95% confidence interval (CI) were used to assess the strength of association between *PTEN* IVS4 polymorphism and cancer risk.

**Results:**

A total of seven case-control studies were finally included in this meta-analysis. The pooled analysis suggested that individuals with *PTEN* IVS4 (−/−) genotype were significantly associated with increased risk of cancer (OR = 1.45, 95% CI = 1.19–1.76, P<0.001) and subgroup of digestive tract cancer (OR = 1.67, 95% CI = 1.28–2.18, P<0.001) compared with (+/+) genotype. The allele analysis revealed that (−) allele was significantly associated with increased risk of cancer (OR = 1.30, 95% CI = 1.12–1.50, P = 0.001) and subgroup of digestive tract cancer (OR = 1.42, 95% CI = 1.16–1.74, P = 0.001) compared with (+) allele. No significant association was observed between *PTEN* IVS4 (+/−) genotype and risk of cancer.

**Conclusion:**

*PTEN* IVS4 (−/−) genotype was significantly associated with increased risk of cancer especially for digestive tract cancer compared with (+/+) genotype. The (−) allele of *PTEN* IVS4 (rs3830675) polymorphism was significantly associated with increased risk of cancer especially for digestive tract cancer compared with (+) allele. The recessive effect model and dominant effect model also demonstrated significant association between *PTEN* IVS4 (rs3830675) polymorphism and increased cancer risk especially for digestive tract cancer. Further large-scale and well-designed studies regarding different ethnicities are still required to confirm the results of our meta-analysis.

## Introduction

The global burden of cancer continues to increase largely with approximately 12.7 million cancer cases and 7.6 million cancer deaths each year worldwide [1]. Carcinogenesis is a multi-step and complex process influenced by various environmental and genetic factors. Emerging evidence has proved that gene polymorphism plays an important role in the difference of individual susceptibilities to cancer [2]. Identification of gene polymorphism that is associated to risk of cancer would largely benefit the early prevention and treatment for cancer.

Phosphatase and tensin homolog (*PTEN*), a well established tumor suppressor gene, is mapped to chromosome 10q23.3. *PTEN* gene spans 105 kb including nine exons and eight introns. The 403 amino-acid PTEN protein is a dual phosphatase, acting at both serine-threonine and tyrosine sites [3]. PTEN protein contains three parts: an N-terminal phosphatase catalytic domain, a C-terminal C2 domain and a 50 amino-acid C-terminal tail which comprises a PDZ binding motif and CK2 phosphorylation sites [4]. PTEN is involved in the regulation of cell growth, proliferation, and apoptosis in signal transduction pathways and participates in the control of cell cycle [5], [6]. Since its first clone in 1997, somatic *PTEN* mutations have been widely reported in various types of cancer including prostate cancer, breast cancer, endometrial cancer and so on [7]–[9]. In recent years, much attention has been paid to the germline polymorphism of *PTEN* gene. *PTEN* polymorphisms have been reported to be involved in multiple cancers, such as breast cancer [10], [11], gastric cancer [12] and colon cancer [13].

*PTEN* IVS4 polymorphism (rs3830675) with ATCTT insertion at 109 bp downstream of exon 4 in intron 4 was one of the common *PTEN* polymorphisms. Most recently, increasing studies investigated the association between *PTEN* IVS4 polymorphism (rs3830675) and risk of various types of cancer including breast cancer [11], [14], colorectal cancer [13], gastric cancer [12], [15], esophageal cancer [16] and prostate cancer [17]. However, the results from individual studies were inconclusive. For instance, Canbay et al. suggested that *PTEN* IVS4 (−/−) genotype was associated with increased risk of colorectal cancer [13]; but George et al. reported that no significant association was observed between *PTEN* IVS4 polymorphism and susceptibility to prostate cancer [17].

Until now, no meta-analysis has been performed to investigate the association of *PTEN* IVS4 polymorphism (rs3830675) with susceptibility to cancer. To explore whether *PTEN* IVS4 polymorphism (rs3830675) was associated with risks of cancer and specific cancer subtypes, we performed a meta-analysis on the association between *PTEN* IVS4 polymorphism (rs3830675) and cancer risk in the present study.

## Materials and Methods

### Identification and Eligibility of Relevant Studies

Electronic databases of Web of Science, PubMed and Chinese National Knowledge Infrastructure (CNKI) were systematically searched using different combinations of the search terms including “phosphatase and tensin homolog/PTEN”, “polymorphism/mutation/variant” and “cancer/neoplasm/malignancy”. The corresponding Chinese search terms were used when searching Chinese database. References cited in each included literatures were further searched manually to identify potential available studies. If the information provided in the literature was not sufficiently clear, the author was contacted for additional raw data. When overlapping data exists, only the study with more complete information was adopted. The last search date was November 12th, 2013.

### Inclusion and Exclusion Criteria

All the included studies must meet the following criteria: case-control study; studies concerning the association between *PTEN* gene IVS4 polymorphism (rs3830675) and risk of cancer; studies published in English or Chinese; studies with sufficient raw data for estimating odds ratios (OR) and their 95% confidence interval (CI). The main reasons for exclusion were: duplicate data; reviews; not relevant to cancer or specific polymorphism; animal experiments; not case-control designed; no raw data even after contacting the author.

### Data Extraction

Two authors (Liping Sun and Jingwei Liu) independently extracted the data from the included studies. The following data were extracted from each individual study: first author, publication year, ethnicity of the studied population, cancer type, numbers of each genotype in cases and controls, genotyping methods for *PTEN* gene IVS4 polymorphism, source of controls. The conflict was resolved after discussion and consensus was finally reached on all of the extracted data.

### Statistical Analysis

All the statistical analysis was performed by Stata software (Version 11.0; StataCorp, College Station, TX). ORs and their 95%CI were used to assess the strength of association between *PTEN* gene polymorphism and cancer risk. P value<0.05 was regarded as statistically significant. Heterogeneity was assessed by I-squared (I^2^) value and using Q statistic (P<0.10 suggests significant heterogeneity between studies) [18]. When heterogeneity between studies was not significant, a fixed-effects model using Mantel-Haenszel method [19] was used to calculate the pooled ORs. Otherwise, a random-effects model using DerSimonian and Laird method [20] was performed. Sensitivity analysis was performed to explore heterogeneity when significant heterogeneity was indicated. Subgroup analyses were performed to explore the effects of ethnicity, cancer type and source of controls. In addition, publication bias were assessed qualitatively by performing funnel plots and evaluated quantitatively by Begg’s test [21] and Egger’s test [22], respectively. P value<0.1 for Begg’s and Egger’s tests suggests significant publication bias.

## Results

### Study Characteristics

**Figure 1:**
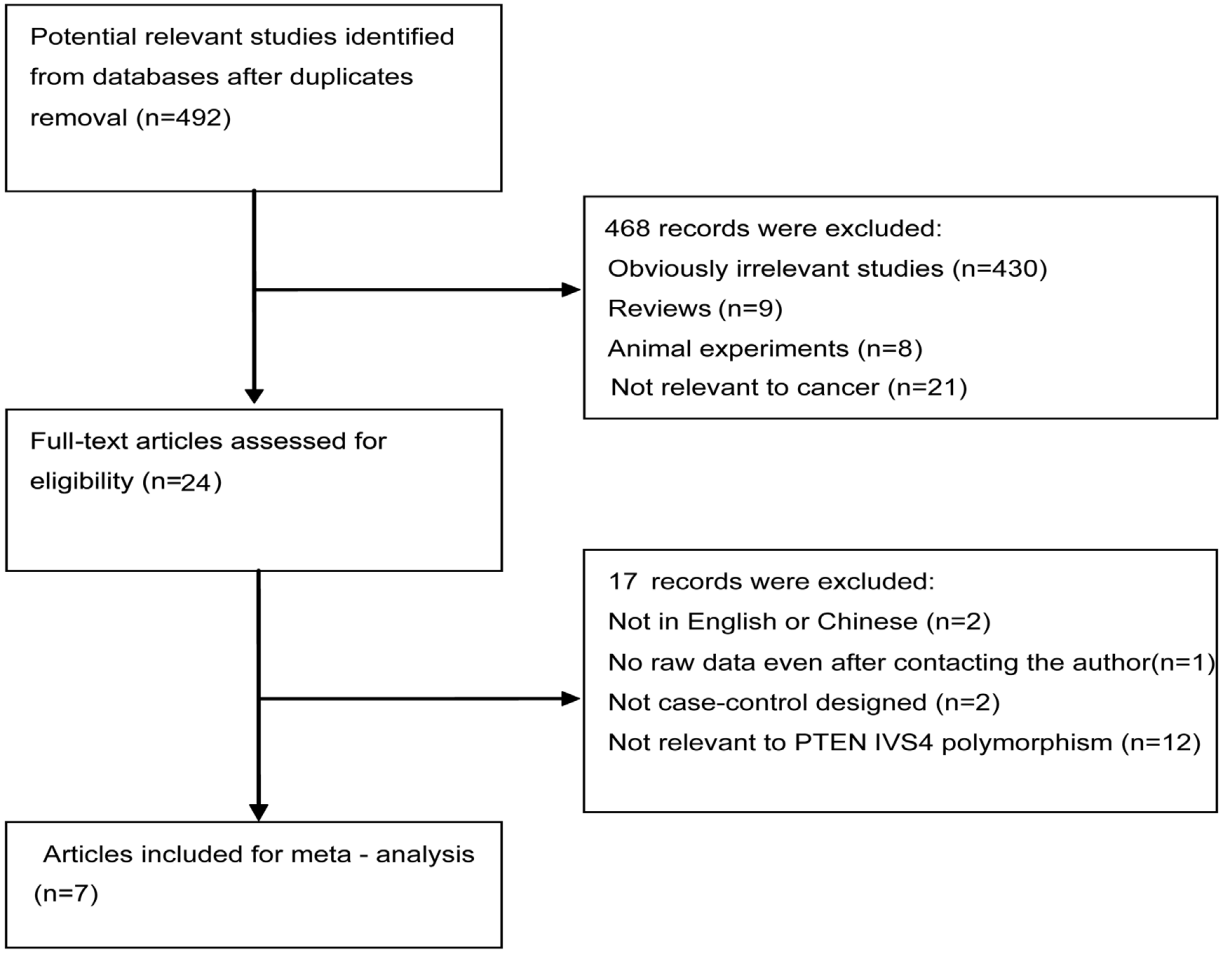
The flowchart of literature inclusion and exclusion.

This meta-analysis was organized based on PRISMA statement (PRISMA Checklist). A total of 492 literatures were obtained from electronic databases after duplicates removal. After reviewing the titles and abstracts, 468 articles were excluded mainly due to no relevance, reviews, animal experiments or not about cancer. Subsequently, the left 24 publications were further evaluated for eligibility. Seventeen literatures were removed because of not in English or Chinese, no raw data, not case-control designed or not concerning IVS4 polymorphism. Finally, seven full-text articles with eligibility were included in the present meta-analysis. The detailed flow chart of study selection was shown in Figure 1.

The baseline characteristics of the studies included in this meta-analysis were summarized in Table 1. All the included case-control studies were published in English or Chinese from 2001 to 2013, altogether consisting of 1831 cases and 2767 controls. The ethnicities of the studied populations covered Turkish, Chinese and American. The *PTEN* IVS4 polymorphism in each study was all detected by polymerase chain reaction-restriction fragment length polymorphism (PCR-RFLP) method. The studied cancer types of the publications consisted of breast cancer, colorectal cancer, gastric cancer, esophageal cancer and prostate cancer. Cancer types including esophageal cancer, gastric cancer and colorectal cancer were integrated as digestive tract cancer when performing subgroup analysis based on cancer type.

**Table 1:** Characteristics of the included studies in this meta-analysis.

					Case				Control				
Author	Year	Ethnicity	Cancertype	Controlssource	N	(+/+)	(+/−)	(−/−)	N	(+/+)	(+/−)	(−/−)	Genotypingmethod
Ozturk, O.	2013	Turkish	Breast cancer	PB	118	6	55	57	128	8	79	41	PCR-RFLP
Canbay, E.	2013	Turkish	Colorectal cancer	PB	203	7	81	115	245	15	144	86	PCR-RFLP
Canbay, E.	2013	Turkish	Gastric cancer	HB	93	7	29	57	113	10	60	43	PCR-RFLP
Zhao, Z, H.	2012	Chinese	Breast cancer	HB	210	63	80	67	210	53	109	48	PCR-RFLP
Ge, H.	2008	Chinese	Gastric cancer	PB	257	48	146	63	634	167	329	138	PCR-RFLP
Ge, H.	2007	Chinese	Esophageal cancer	PB	350	73	183	94	634	167	329	138	PCR-RFLP
George, D. J.	2001	American	Prostate cancer	PB	600	57	281	262	803	90	367	346	PCR-RFLP

Abbreviations: PB, population-based; HB, hospital-based.

### Quantitative Data Synthesis

**Figure 2:**
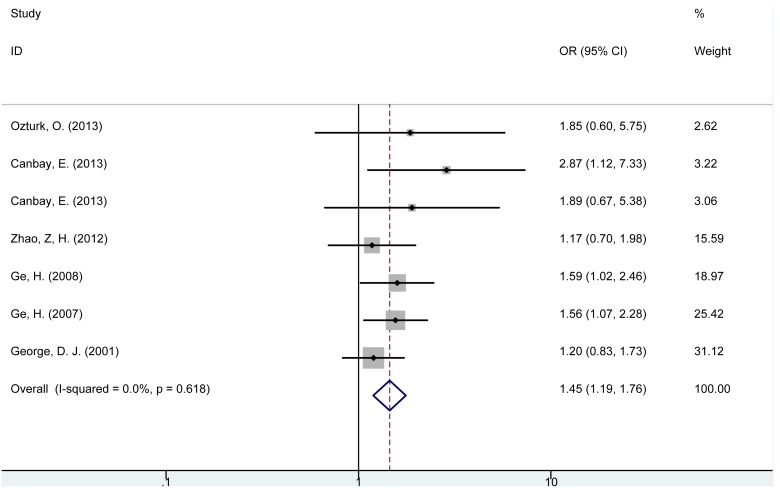
Forest plot for the association between *PTEN* IVS4 (rs3830675) polymorphism and cancer risk (−/− vs. +/+).

The results of the quantitative synthesis of the data were summarized in Table 2. Individuals with *PTEN* IVS4 (−/−) genotype were significantly associated with increased risk of cancer (OR = 1.45, 95% CI = 1.19–1.76, P<0.001, Figure 2) compared with (+/+) genotype. For subgroup analysis according to cancer type, *PTEN* IVS4 (−/−) genotype was observed to be associated with increased risk of digestive tract cancer (OR = 1.67, 95% CI = 1.28–2.18, P<0.001) but no significant association was found with breast cancer (OR = 1.27, 95% CI = 0.79–2.04, P = 0.318) or prostate cancer (OR = 1.20, 95% CI = 0.83–1.73, P = 0.342). As for different ethnicities, carriers of *PTEN* IVS4 (−/−) genotype were significantly associated with increased risk of cancer in Turkish and Chinese, but not in American population. In the subgroup analysis based on source of control, increased cancer risk was observed in population-based subgroup rather than hospital-based subgroup.

**Table 2:** Meta-analysis results of the association between *PTEN* IVS4 polymorphism (rs3830675) and cancer risk.

Genetic model	Group/Subgroup	N	Heterogeneity Test			Test for overall effect	
			I^2^ (%)	P_het_	Statistical model	OR (95%CI)	P
(−/−) vs. (+/+)	Overall	7	0.00%	0.618	F	**1.45(1.19–1.76)**	**<0.001**
	Digestive tract cancer	4	0.00%	0.682	F	**1.67(1.28–2.18)**	**<0.001**
	Breast cancer	2	0.00%	0.473	F	1.27(0.79–2.04)	0.318
	Turkish	3	0.00%	0.790	F	**2.23(1.24–4.03)**	**0.008**
	Chinese	3	0.00%	0.629	F	**1.47(1.14–1.89)**	**0.003**
	PB	5	0.00%	0.477	F	**1.49(1.20–1.85)**	**<0.001**
	HB	2	0.00%	0.422	F	1.29(0.81–2.06)	0.279
(+/−) vs. (+/+)	Overall	7	43.90%	0.098	R	1.10(0.84–1.43)	0.484
	Digestive tract cancer	4	0.00%	0.536	F	**1.33(1.05–1.68)**	**0.016**
	Breast cancer	2	0.00%	0.508	F	0.66(0.43–1.01)	0.054
	Turkish	3	0.00%	0.743	F	0.95(0.52–1.71)	0.851
	Chinese	3	79.10%	0.008	R	1.09(0.67–1.78)	0.732
	PB	5	0.00%	0.853	F	**1.31(1.07–1.59)**	**0.007**
	HB	2	0.00%	0.850	F	**0.63(0.41–0.96)**	**0.033**
(−/−) vs. [(−/+) and (+/+)]	Overall	7	74.70%	0.001	R	**1.56(1.19–2.05)**	**0.001**
	Digestive tract cancer	4	75.10%	0.007	R	**1.70(1.16–2.49)**	**0.007**
	Breast cancer	2	0.00%	0.511	F	**1.74(1.24–2.42)**	**0.001**
	Turkish	3	0.00%	0.767	F	**2.32(1.77–3.04)**	**<0.001**
	Chinese	3	0.00%	0.558	F	**1.32(1.08–1.61)**	**0.007**
	PB	5	77.60%	0.001	R	**1.45(1.06–1.98)**	**0.020**
	HB	2	44.80%	0.178	F	**1.90(1.35–2.67)**	**<0.001**
[(−/−) and (−/+)] vs. (+/+)	Overall	7	12.50%	0.334	F	**1.26(1.06–1.49)**	**0.008**
	Digestive tract cancer	4	0.00%	0.871	F	**1.45(1.16–1.81)**	**0.001**
	Breast cancer	2	0.00%	0.444	F	0.84(0.56–1.25)	0.385
	Turkish	3	0.00%	0.794	F	1.44(0.81–2.54)	0.217
	Chinese	3	67.50%	0.046	R	1.21(0.84–1.75)	0.313
	PB	5	0.00%	0.839	F	**1.38(1.14–1.66)**	**0.001**
	HB	2	0.00%	0.458	F	0.84(0.57–1.25)	0.385
(−) allele vs. (+) allele	Overall	7	60.00%	0.020	R	**1.30(1.12–1.50)**	**0.001**
	Digestive tract cancer	4	58.00%	0.067	R	**1.42(1.16–1.74)**	**0.001**
	Breast cancer	2	41.80%	0.190	F	1.21(0.97–1.51)	0.089
	Turkish	3	0.00%	0.699	F	**1.71(1.39–2.10)**	**<0.001**
	Chinese	3	0.00%	0.724	F	**1.20(1.06–1.36)**	**0.003**
	PB	5	64.00%	0.025	R	**1.29(1.09–1.53)**	**0.003**
	HB	2	74.00%	0.050	R	1.37(0.83–2.26)	0.220

Abbreviations: R, random effect model; F, fixed effect model; PB, population-based; HB, hospital-based.

**Figure 3:**
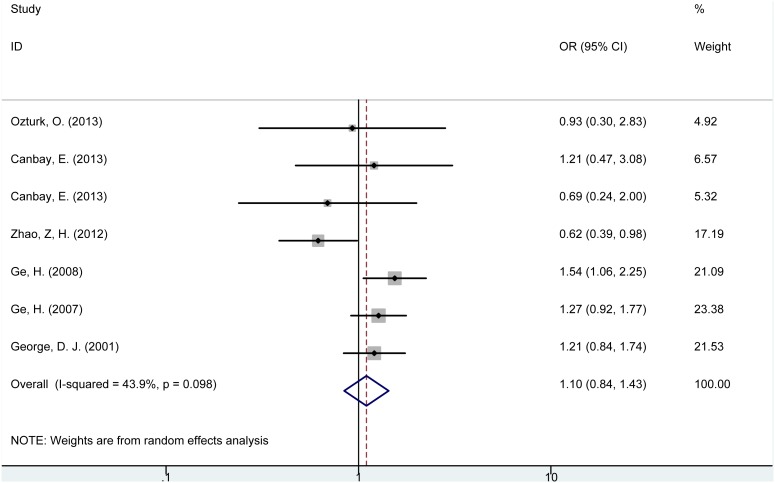
Forest plot for the association between *PTEN* IVS4 (rs3830675) polymorphism and cancer risk (+/− vs. +/+).

Individuals with *PTEN* IVS4 (+/−) genotype were not significantly associated with risk of cancer (OR = 1.10, 95% CI = 0.84–1.43, P = 0.484, Figure 3) compared with (+/+) genotype. However, subgroup analysis based on different cancer type indicated that *PTEN* IVS4 (+/−) genotype was significantly related with increased risk of digestive tract cancer (OR = 1.33, 95% CI = 1.05–1.68, P = 0.016); while no such association was detected in subgroups of breast cancer or prostate cancer. In addition, no significant association was observed in different subgroup based on ethnicities. It was worth noting that subgroup analysis of population-based (PB) and hospital-based (HB) demonstrated controversial outcomes (PB: OR = 1.31, 95% CI = 1.07–1.59, P = 0.007; HB: OR = 0.63, 95% CI = 0.41–0.96, P = 0.033), which suggested that the selection of the controls might influence the result of the relation between *PTEN* IVS4 (+/−) genotype and cancer risk.

The recessive effect model and dominant effect model also demonstrated significant association between *PTEN* IVS4 (rs3830675) polymorphism and increased cancer risk (−/− vs. (−/+ and +/+): OR = 1.56, 95% CI = 1.19–2.05, P = 0.001, Figure S2; (−/+ and −/−) vs. (+/+): OR = 1.26, 95% CI = 1.06–1.49, P = 0.008, Figure S3) especially for digestive tract cancer (−/− vs. (−/+ and +/+): OR = 1.70, 95% CI = 1.16–2.49, P = 0.007; (−/+ and −/−) vs. (+/+): OR = 1.45, 95% CI = 1.16–1.81, P = 0.001).

**Figure 4:**
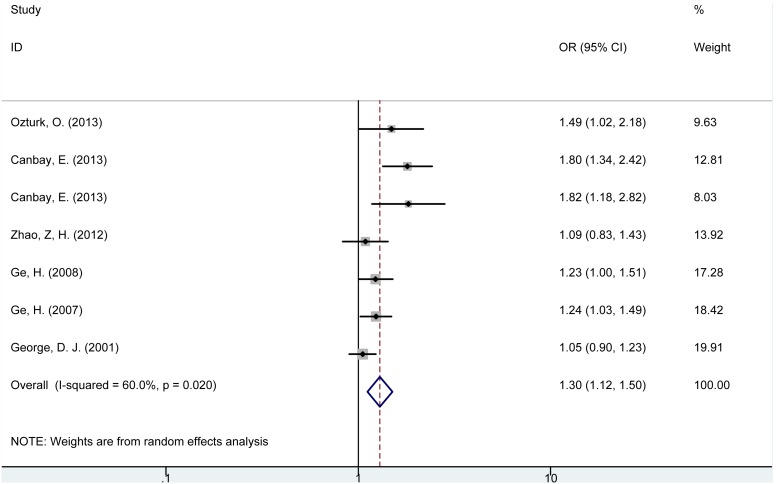
Forest plot for the association between *PTEN* IVS4 (rs3830675) polymorphism and cancer risk (−allele vs. +allele).

The allele analysis revealed that (−) allele of *PTEN* IVS4 (rs3830675) polymorphism was significantly associated with increased risk of cancer (OR = 1.30, 95% CI = 1.12–1.50, P = 0.001, Figure 4) and digestive tract cancer (OR = 1.42, 95% CI = 1.16–1.74, P = 0.001) compared with (+) allele. No such significant association was found in subgroups of breast cancer or prostate cancer. In stratified analysis according to different ethnicities, consistently increased risk of cancer for *PTEN* IVS4 (−) allele was observed in Turkish and Chinese populations, but no significant relation was found in American.

### Heterogeneity Test, Sensitivity Analysis and Publication Bias

In the comparison of *PTEN* IVS4 (−/−) genotype with (+/+) genotype, no significant heterogeneity was observed in the overall analysis and all of the subgroup analyses. Therefore, fix-effect model was adopted. In several comparisons of *PTEN* IVS4 (+/−) genotype with (+/+) genotype and allele analysis, random-effect model were used due to significant heterogeneity (Table 2). Sensitivity analysis was subsequently performed to detect the influence of individual study on the pooled estimate by omitting one study from the pooled analysis each time. The exclusion of each single study did not significantly change the pooled OR (figure not shown), suggesting that the results of the meta-analysis were robust.

The Begg’s test and Egger’s test were performed to evaluate the publication bias of the studies quantitatively. The detailed information for publication bias test was shown in Table 3. Significant publication bias were observed in the allele analysis and recessive effect model analysis of *PTEN* gene IVS4 (rs3830675) polymorphism. Besides, funnel plot that qualitatively assessed the publication bias of association between *PTEN* IVS4 (−/−) genotype and cancer risk was presented in Figure S1.

**Table 3:** Results of publication bias test.

Compared genotype	Begg’s test		Egger’s test	
	z value	P value	t value	P value
(−/−) vs. (+/+)	1.05	0.293	1.75	0.141
(+/−) vs. (+/+)	−1.35	0.176	−0.96	0.383
(−/−) vs. [(−/+) and (+/+)]	1.95	0.051	3.59	0.016
[(−/−) and (−/+)] vs. (+/+)	−0.15	0.881	0.00	0.996
(−) allele vs. (+) allele	1.65	0.099	2.66	0.045

## Discussion

Results of previous studies concerning the relationship of *PTEN* gene IVS4 (rs3830675) polymorphism with cancer risk turn out to be controversial [11]–[17]. To our best knowledge, this was the first meta-analysis investigating the role of *PTEN* IVS4 polymorphism in carcinogenesis. By performing the current meta-analysis, we suggested that *PTEN* IVS4 (−/−) genotype and the (−) allele were significantly associated with increased risk of cancer especially for digestive tract cancer, respectively. However, no significant association was observed between *PTEN* IVS4 (+/−) genotype and risk of cancer.

After its first identification as a tumor suppressor gene in 1997, the role of *PTEN* gene in carcinogenesis has attracted special interest. Somatic mutations have been detected in a variety of cancer including breast, prostate, melanoma and endometrial [23], making it one of the most frequently mutated tumor suppressors. In addition, germline mutations of *PTEN* predispose carriers to develop Cowden’s disease and Bannayan-Zonana syndrome [24], [25]. Recently, the association between polymorphisms of *PTEN* gene and risk of cancer has been investigated in various types of cancer. One of the most commonly studied polymorphism was *PTEN* gene IVS4 (rs3830675) polymorphism in intron 4. As individual studies demonstrated inconsistent results, we performed the present meta-analysis to elucidate the exact role of *PTEN* gene IVS4 (rs3830675) polymorphism in carcinogenesis.

By performing the current meta-analysis, we found that *PTEN* IVS4 (−/−) genotype was significantly associated with increased risk of cancer (OR = 1.45) and subgroup of digestive tract cancer (OR = 1.67) compared with (+/+) genotype; no significant association was observed between (−/−) genotype and risks of breast cancer or prostate cancer. The recessive effect model and dominant effect model also demonstrated significant association between *PTEN* IVS4 (rs3830675) polymorphism and increased cancer risk (−/− vs. (−/+ and +/+): OR = 1.56; (−/+ and −/−) vs. (+/+): OR = 1.26) especially for digestive tract cancer. Similarly, (−) allele of *PTEN* IVS4 (rs3830675) polymorphism was also significantly associated with increased risk of cancer (OR = 1.30) and digestive tract cancer (OR = 1.42) compared with (+) allele; no such significant association was found in subgroups of breast cancer or prostate cancer. Different cancer has its distinct mechanisms of initiation and progression, the obvious different outcome of diverse cancer subtypes in this meta-analysis indicated that *PTEN* IVS4 (rs3830675) polymorphism might confer altered risks to various types of cancer. The consistent association between *PTEN* IVS4 (rs3830675) polymorphism and risk of digestive tract cancer in different genetic models suggested that *PTEN* might have specific role in the carcinogenesis of digestive tract cancer, which requires further studies to explore. As for different ethnicities, *PTEN* IVS4 (−/−) genotype and (−) allele were associated with increased risk of cancer in both Turkish and Chinese but not in American. Although different associations were observed in Asian and Caucasian, conclusion based on different ethnicities should be drawn carefully due to the limited study number for Caucasians. In addition, heterozygous (+/−) genotype was not significantly associated with altered cancer risk according to the pooled estimate in this meta-analysis.

The role of *PTEN* as a tumor suppressor has been firmly established. PTEN antagonizes the phosphoinositol-3-kinase/AKT signaling pathway and suppresses cell survival as well as cell proliferation, thereby safeguards important cellular machineries against tumorigenesis [26]. Besides, PTEN controls a variety of biological processes including cell proliferation, growth, migration and death [27]. Previous investigations regarding *PTEN* polymorphisms mainly focused on the exon region of *PTEN* gene. Quite recently, the intron polymorphism of *PTEN* drew increasing attention. Although introns were originally believed to be nonfunctional because they do not code for proteins, it has been suggested that some of these sequences possess crucial functions [28]. Some introns could regulate expression of gene [29] while other ones could be further processed after splicing to generate noncoding RNA molecules [30]. Important intron sites with polymorphism could disrupt splicing process during transcription. The *PTEN* IVS4 (rs3830675) polymorphism may lead to a splicing error or may be by linkage disequilibrium with another locus to affect the expression and function of the *PTEN*. The alternation of *PTEN* expression would inevitably change the role of *PTEN* in maintaining genome stability, and loss of function of this tumor suppressor might therefore leads to carcinogenesis. Although the above-mentioned possible mechanism might partially explain the observed association between *PTEN* IVS4 (rs3830675) polymorphism and cancer susceptibilities, rare functional study has been carried out and the exact mechanism remain largely elusive. Future functional studies are still needed to clarify the exact mechanism of the role of *PTEN* IVS4 (rs3830675) polymorphism in carcinogenesis.

Several limitations should be acknowledged in this meta-analysis when interpreting the results. First, the sample size of this meta-analysis was not sufficiently large especially for the subgroup analyses. Second, only the studies published in English or Chinese were included in this meta-analysis, which might cause publication bias. Third, the ethnicities of most studies were Asian populations with only one study carried out in Caucasian, which deserves further confirmations in other ethnicities. Finally, because other important data such as sex, age, family history and environment risk factors were not available, we could not obtained results with adjustments by other co-variables.

### Conclusion

To be concluded, this meta-analysis indicated that *PTEN* IVS4 (−/−) genotype was significantly associated with increased risk of cancer especially for digestive tract cancer compared with (+/+) genotype. The (−) allele of *PTEN* IVS4 (rs3830675) polymorphism was significantly associated with increased risk of cancer especially for digestive tract cancer compared with (+) allele. The recessive effect model and dominant effect model also demonstrated significant association between *PTEN* IVS4 (rs3830675) polymorphism and increased cancer risk especially for digestive tract cancer. Further large-scale and well-designed studies regarding different ethnicities are still required to confirm the results of our meta-analysis.

## Supporting Information

Figure S1 **Funnel plot for studies of association between** ***PTEN*** **IVS4 (rs3830675) polymorphism and cancer risk (−/− vs. +/+).**
(TIF)

Figure S2 **Funnel plot for studies of association between** ***PTEN*** **IVS4 (rs3830675) polymorphism and cancer risk (−/− vs. [−/+ and +/+]).**
(TIF)

Figure S3 **Funnel plot for studies of association between** ***PTEN*** **IVS4 (rs3830675) polymorphism and cancer risk ([−/− and −/+] vs. +/+).**
(TIF)

Checklist S1 **PRISMA checklist.**
(DOC)
