# Supplementary figures and images for: Association between PTEN Gene IVS4 Polymorphism and Risk of Cancer: A Meta-Analysis
Source: PLoS One. 2014 Jun 5;9(6):e98851. doi: 10.1371/journal.pone.0098851 (PMC4047023; doi:10.1371/journal.pone.0098851)

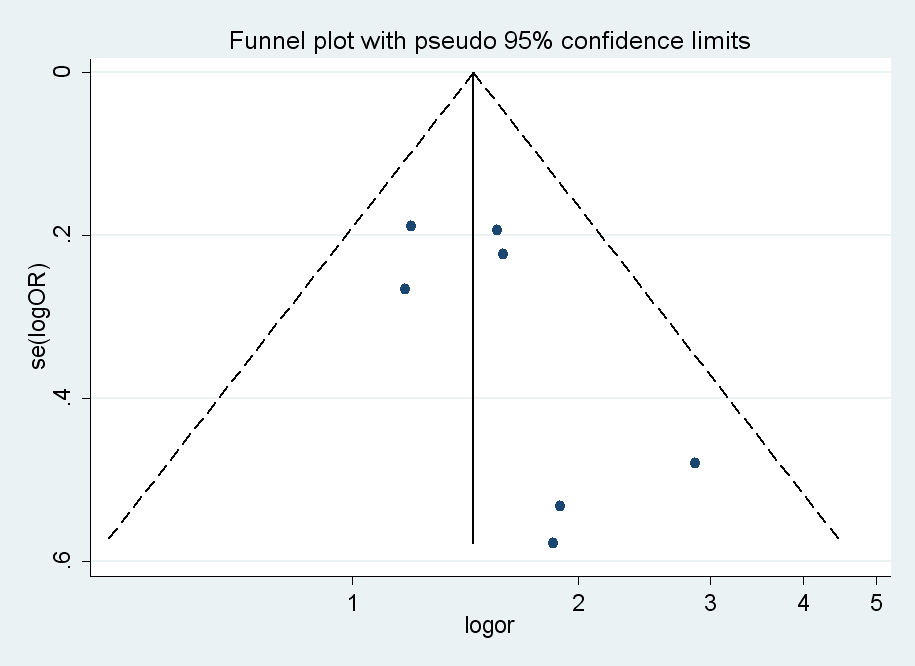

Supplement: Figure S1 — Funnel plot for studies of association between PTEN IVS4 (rs3830675) polymorphism and cancer risk (−/− vs. +/+). (TIF) [file pone.0098851.s001.tif]

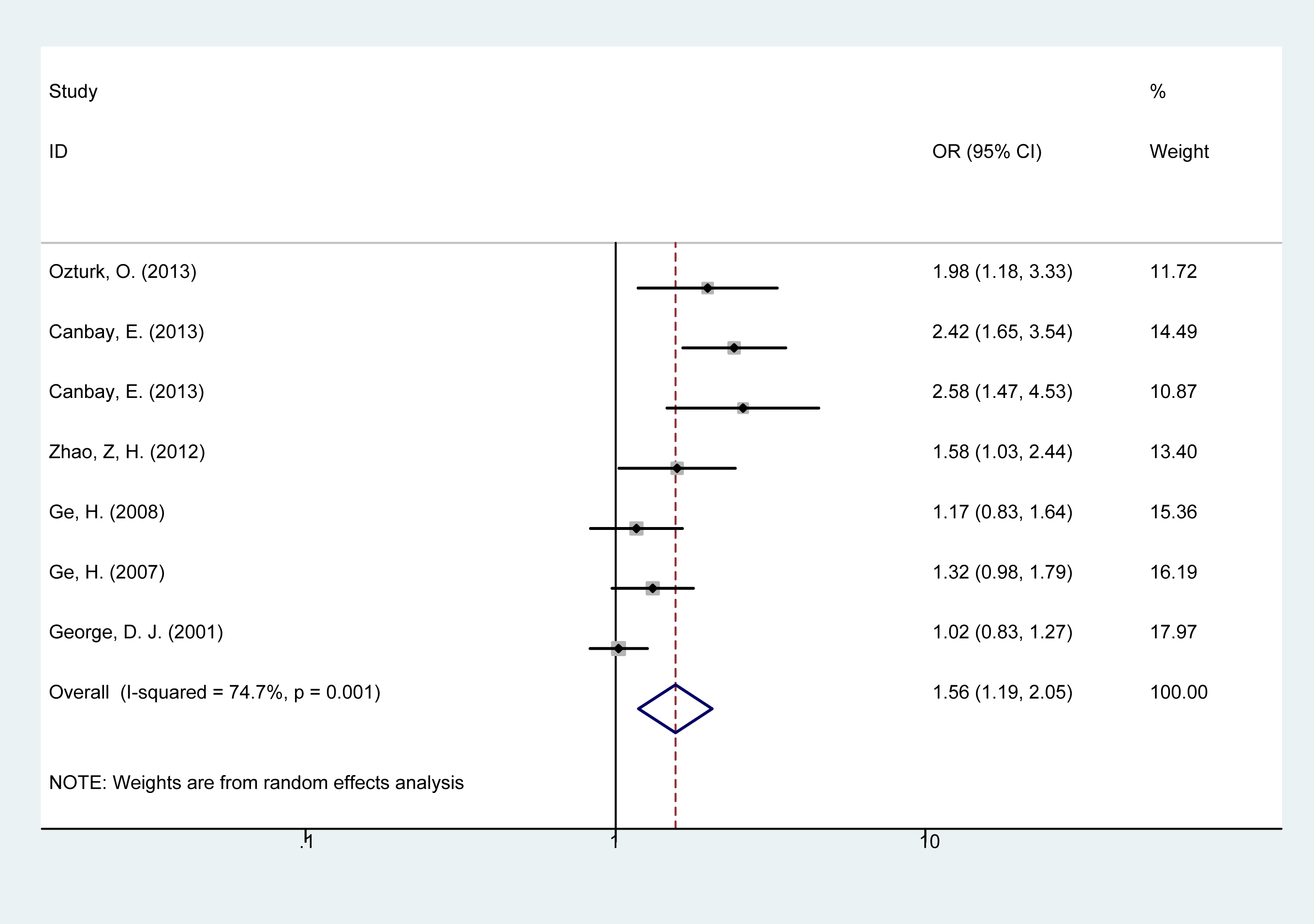

Supplement: Figure S2 — Funnel plot for studies of association between PTEN IVS4 (rs3830675) polymorphism and cancer risk (−/− vs. [−/+ and +/+]). (TIF) [file pone.0098851.s002.tif]

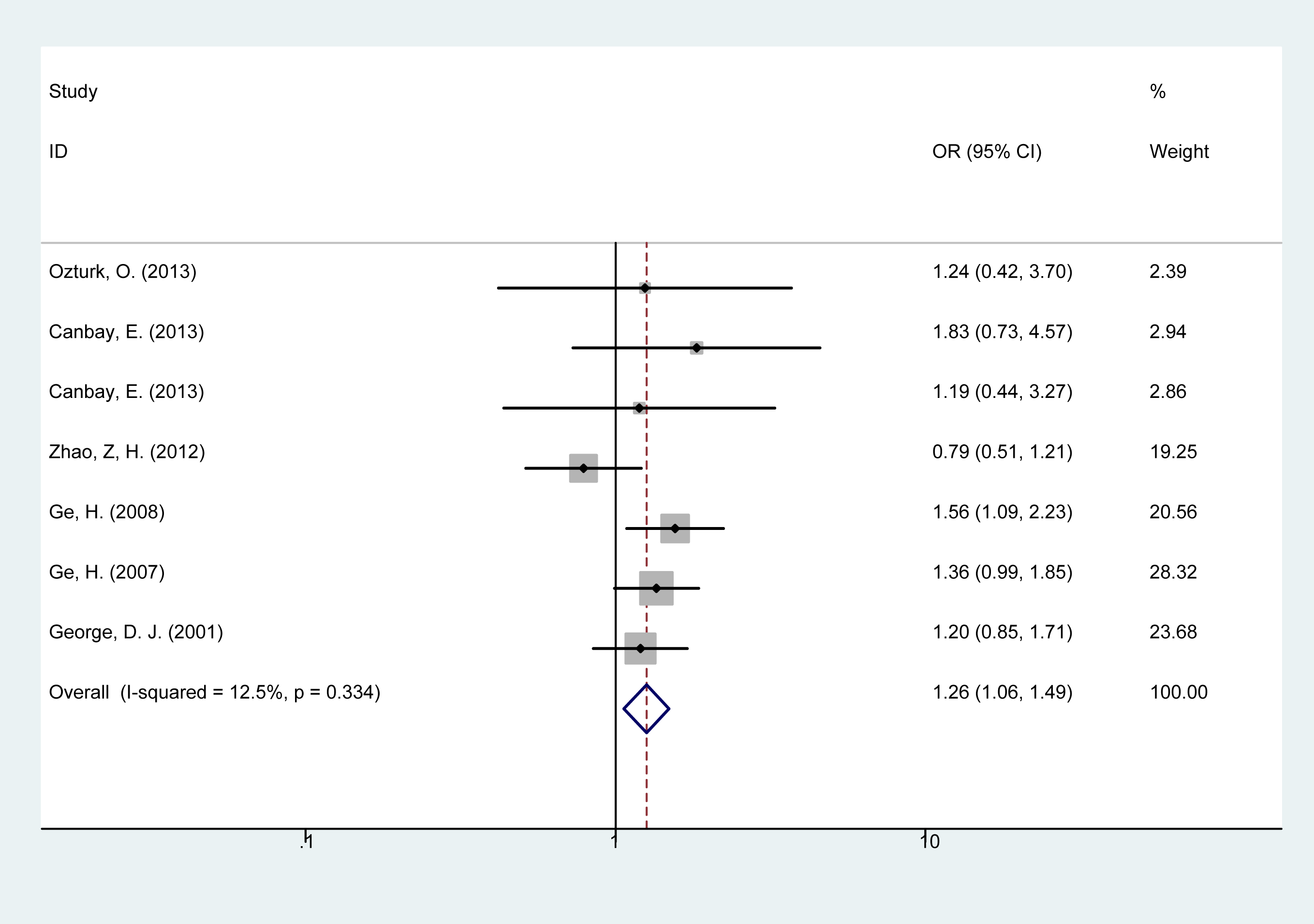

Supplement: Figure S3 — Funnel plot for studies of association between PTEN IVS4 (rs3830675) polymorphism and cancer risk ([−/− and −/+] vs. +/+). (TIF) [file pone.0098851.s003.tif]
